# Genotoxic mechanisms for the carcinogenicity of the environmental pollutants and carcinogens *o*-anisidine and 2-nitroanisole follow from adducts generated by their metabolite *N*-(2-methoxyphenyl)-hydroxylamine with deoxyguanosine in DNA

**DOI:** 10.2478/v10102-009-0004-4

**Published:** 2009-03

**Authors:** Marie Stiborová, Karel Naiman, Markéta Martínková, Václav Martínek, Martina Svobodová, Heinz H. Schmeiser, Eva Frei

**Affiliations:** 1 Department of Biochemistry, Faculty of Science, Charles University, Prague, Albertov 2030, 128 40 Prague 2, Czech Republic; 2 German Cancer Research Center, Im Neuenheimer Feld 280, 69120 Heidelberg, Germany

**Keywords:** carcinogens, *o*-anisidine, 2-nitroanisole, *N*-(2-methoxyphenyl)hydroxylamine, metabolism, DNA adducts, cancer

## Abstract

An aromatic amine, *o*-anisidine (2-methoxyaniline) and its oxidative counterpart, 2-nitroanisole (2-methoxynitrobenzene), are the industrial and environmental pollutants causing tumors of the urinary bladder in rats and mice. Both carcinogens are activated to the same proximate carcinogenic metabolite, *N*-(2-methoxyphenyl)hydroxylamine, which spontaneously decomposes to nitrenium and/or carbenium ions responsible for formation of deoxyguanosine adducts in DNA *in vitro* and *in vivo*. In other words, generation of *N*-(2-methoxyphenyl)hydroxylamine is responsible for the genotoxic mechanisms of the o-anisidine and 2-nitroanisole carcinogenicity. Analogous enzymes of human and rat livers are capable of activating these carcinogens. Namely, human and rat cytochorme P4502E1 is the major enzyme oxidizing *o*-anisidine to *N*-(2-methoxyphenyl)hydroxylamine, while xanthine oxidase of both species reduces 2-nitroanisole to this metabolite. Likewise, *O*-demethylation of 2-nitroanisole, which is the detoxication pathway of its metabolism, is also catalyzed by the same human and rat enzyme, cytochorme P450 2E1. The results demonstrate that the rat is a suitable animal model mimicking the fate of both carcinogens in humans and suggest that both compounds are potential carcinogens also for humans.

## Introduction

**Figure 1:**
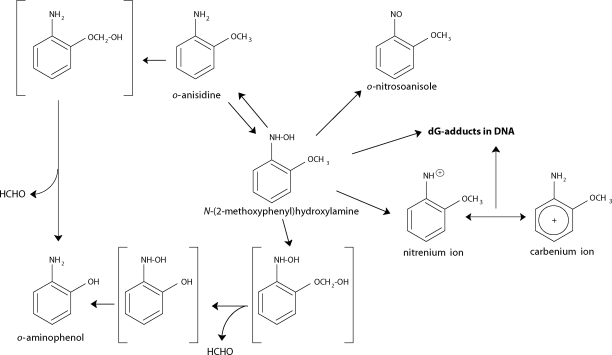
Pathways of o-anisidine metabolism by the cytochrome P450 system showing the characterized metabolites and those proposed to form DNA adducts. The compounds shown in brackets were not detected under the experimental conditions.

**Figure 2:**
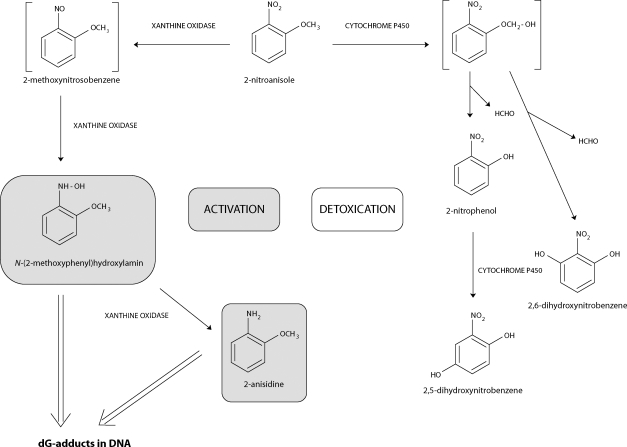
Pathways of 2-nitroanisole metabolism showing the characterized metabolites and those proposed to form DNA adducts. The compounds shown in brackets were not detected under the experimental conditions.

Aromatic nitro-compounds and amines are potent toxic or carcinogenic compounds, presenting a considerable danger to the human population (NTP, 1978; 1993; Garner et al., 1984; IARC, 1982;1989). They are widely distributed environmental pollutants found in workplaces (e.g. in chemical industry), in emissions from diesel and gasoline engines and on the surface of ambient air particulate matter (IARC, 1989; 1982; NTP, 1978; 1993), where they add to local and regional pollution (car exhausts, technological spills). The toxicity and carcinogenicity of these compounds, their metabolic pathways and the persistence of residues of these compounds and/or their metabolites in organisms have been examined (NTP, 1978; 1993; IARC, 1989; 1982; Purohit and Basu, 2000). However, the knowledge of the fate of several aromatic nitro compounds or aromatic amines and their physiological effects in humans is still scarce (Purohit and Basu, 2000). This also the case of compounds such as 2-methoxyaniline (*o*-anisidine) and 2-nitroanisole (2-methoxynitrobenzene) (Figures 1 and 2).

## Toxicity, carcinogenicity and metabolism of *o*-anisidine and 2-nitroanisole

*o*-Anisidine and 2-nitroanisole are potent carcinogens, causing tumors of the urinary bladder in both genders of rats and mice (NTP, 1978; IARC, 1982; NTP, 1993). The International Agency for Research on Cancer (IARC) has classified *o*-anisidine as a group 2B carcinogens (IARC, 1982), which is possibly carcinogenic to humans. Besides its carcinogenicity it exhibits other toxic effects, including hematological changes, anemia and nephrotoxicity (NTP, 1978; IARC, 1982). *o*-Anisidine is used as an intermediate in the manufacturing of a number of azo and naphthol pigments and dyes, which are used for printing (90%) and for paper (3%) and textile (7%) dyeing (NTP, 1978; Garner *et al*., 1984). Such a wide use of this aromatic amine could result in occupational exposure. Furthermore, it may be released from textiles and leather goods colored with these azo dyes and a large part of the population may be exposed. This carcinogen is also a constituent of cigarette smoke (IARC, 1982; Stabbert *et al*., 2003). This strongly suggests that *o*-anisidine ranks not only among occupational pollutants produced in the manufacturing of chemicals, but also among environmental pollutants; it can be assumed that human exposure is widespread. Indeed, *o*-anisidine was found in human urine samples in the general population, in concentrations of 0.22 µg/l (median) (Weiss and Angerer, 2003). In addition, hemoglobin adducts of *o*-anisidine were detected in blood samples of persons living in urban or rural areas of Germany (Falter *et al*., 1994; Branner *et al*., 1998; Richter *et al*., 2001). The adducts as well as *o*-anisidine in urine might originate not only from the sources mentioned above, but also from a possible *o*-anisidine precursor, 2-methoxynitrobenzene (2-nitroanisole). This chemical was released into the environment in the course of an accident in a German chemical plant, causing subsequently local and regional contamination (Falter *et al*., 1994; Hauthal, 1993; Traupe *et al*., 1997). 2-Nitroanisole exhibits strong carcinogenic activity, causing neoplastic transformation in the urinary bladder, and to a lesser extent, in the spleen, liver and kidneys in rodents (NTP 1993). It is also a toxic compound, causing anemia. The anemia is characterized by increased levels of methemoglobin and accelerated destruction of erythrocytes (NTP 1993).

Recently, we have found that *o*-anisidine is oxidatively activated by peroxidase and cytochrome P450 (CYP) to species binding to DNA *in vitro* (Stiborová *et al*., 2001, 2002; 2005; Rýdlová *et al*., 2005; Naiman *et al*., 2008b). We also demonstrated that *o*-anisidine forms DNA adducts *in vivo*. The same adducts as found in DNA incubated with *o*-anisidine and human microsomes *in vitro* were detected in urinary bladder, the target organ, and to a lesser extent, in liver, kidney and spleen of rats treated with *o*-anisidine (Stiborová *et al*., 2005). The *o*-anisidine-derived DNA adducts were identified as deoxyguanosine adducts formed from a metabolite of *o*-anisidine, *N*-(2-methoxyphenyl)hydroxylamine, which is generated by oxidation of *o*-anisidine with human, rabbit and rat hepatic microsomes (Stiborová *et al*., 2005; Rýdlová *et al*., 2005; Naiman *et al*., 2008b). The same deoxyguanosine adducts were also detected in DNA of the urinary bladder, kidney, liver and spleen of rats treated with 2-nitroanisole (Stiborová *et al*., 2004), an oxidized counterpart of *o*-anisidine, and in DNA incubated with 2-nitroanisole *in vitro* with human and rat hepatic cytosolic enzymes and xanthine oxidase (Stiborová *et al*., 1998; 2004). These enzymatic systems were found to produce *N*-(2-methoxyphenyl)hydroxylamine after 2-nitroanisole reduction (Mikšanová *et al*., 2004a) (Figure 2). The data indicate that formation of *N*-(2-methoxyphenyl)hydroxylamine, the reactive metabolite of both carcinogens, is critical for generation of DNA lesions in target organs. Therefore, it is clear that *N*-(2-methoxyphenyl)hydroxylamine formation and its further conversion, as well as the enzymes participating in such processes, play a key role in carcinogenic effects of both carcinogens. Oxidation of 2-nitroanisole by rat, rabbit and human hepatic microsomal cytochrome P450 enzymes leads to its detoxication. *O*-Demethylation of 2-nitroanisole to 2-nitrophenol and its hydroxylated products, 2,5-dihydroxynitrobenzene and 2,6-dihydroxynitrobenzene, did not form any adducts with DNA (Mikšanová *et al*., 2004b; Dračínská *et al*., 2006). 2-Nitroanisole metabolite 2-nitrophenol is the major metabolite generated by rabbit and rat microsomal enzymes, but 2,5-dihydroxynitrobenzene is the predominant product formed in human microsomal cytochromes P450 (Mikšanová *et al*., 2004b; Dračínská *et al*., 2006; Svobodová *et* al., 2008) (Figure 2). Therefore, hepatic microsomal P450 enzymes participate in detoxication of this environmental carcinogen.

Recently, we have found that *o*-anisidine is oxidized by human, rat and rabbit hepatic microsomes containing cytochromes P450 not only to *N*-(2-methoxyphenyl)hydroxylamine, but that this compound is a subject of complex redox cycling reactions, forming also *o*-aminophenol, 2-nitrosoanisole and one additional metabolite, the exact structure of which has not been identified as yet (Stiborová *et al*., 2005; Naiman *et al*., 2008a; 2008b). *N*-(2-methoxyphenyl)hydroxylamine might also be a subject of complex reactions, and its fate is dependent on the environment, in which it occurs. It can be further metabolized to *o*-aminophenol, *o*-nitrosoanisole and parental *o*-anisidine (Naiman *et al*., 2008a; 2008b), or when nucleophiles such as DNA or proteins are present in the cell, form the adducts (Stiborová *et al*., 2005) (Figure 1).

## Conclusion

The results of our former (Stiborová *et al*., 1998; 2004; 2005) and the present studies (unpublished data) indicate that three DNA adducts found *in vitro* and *in vivo* are formed from *N*-(2-methoxyphenyl)hydroxylamine and deoxyguanosine in DNA. The physico-chemical properties of these adducts suggest that they are polar compounds, in which only one benzene ring of the original *o*-anisidine and/or 2-nitroanisole molecules is bound to this deoxynucleoside. Recently, their structures were suggested to be the structures of adducts, in which nitrenium and/or carbenium ions formed from *N*-(2-methoxyphenyl)hydroxylamine are bound to C8, O^6^, and/or N^2^ in the guanine residue (Stiborová *et al*., 2005). The aim of our current and future research in our laboratory is to confirm this suggestion. Procedures to prepare the adducts generated by reactions *N*-(2-methoxyphenyl)hydroxylamine with deoxygunaosine and HPLC methods to separate adducts from other components of the reaction mixtures were developed in our laboratory. They will lead to obtain adducts in amounts sufficient for their structural characterization by mass- and NMR spectrometry.
